# Chronic Brain Imaging Across a Transparent Nanocrystalline Yttria-Stabilized-Zirconia Cranial Implant

**DOI:** 10.3389/fbioe.2020.00659

**Published:** 2020-06-30

**Authors:** David L. Halaney, Carrie R. Jonak, Junze Liu, Nami Davoodzadeh, Mildred S. Cano-Velázquez, Pasha Ehtiyatkar, Hyle Park, Devin K. Binder, Guillermo Aguilar

**Affiliations:** ^1^Laboratory of Guillermo Aguilar, Department of Mechanical Engineering, University of California, Riverside, Riverside, CA, United States; ^2^Laboratory of Devin Binder, Division of Biomedical Sciences, University of California, Riverside, Riverside, CA, United States; ^3^Laboratory of Hyle Park, Department of Bioengineering, University of California, Riverside, Riverside, CA, United States; ^4^Laboratory of Juan Hernandez-Cordero, Instituto de Investigaciones en Materiales, Universidad Nacional Autónoma de México, Mexico City, Mexico; ^5^Department of Neuroscience, University of California, Riverside, Riverside, CA, United States

**Keywords:** cranial implant, window to the brain, brain, imaging, optical coherence tomography, laser speckle imaging

## Abstract

Repeated non-diffuse optical imaging of the brain is difficult. This is due to the fact that the cranial bone is highly scattering and thus a strong optical barrier. Repeated craniotomies increase the risk of complications and may disrupt the biological systems being imaged. We previously introduced a potential solution in the form of a transparent ceramic cranial implant called the Window to the Brain (WttB) implant. This implant is made of nanocrystalline Yttria-Stabilized Zirconia (nc-YSZ), which possesses the requisite mechanical strength to serve as a permanent optical access window in human patients. In this present study, we demonstrate repeated brain imaging of *n* = 5 mice using both OCT and LSI across the WttB implant over 4 weeks. The main objectives are to determine if the WttB implant allows for chronic OCT imaging, and to shed further light on the question of whether optical access provided by the WttB implant remains stable over this duration in the body. The Window to the Brain implant allowed for stable repeated imaging of the mouse brain with Optical Coherence Tomography over 28 days, without loss of signal intensity. Repeated Laser Speckle Imaging was also possible over this timeframe, but signal to noise ratio and the sharpness of vessels in the images decreased with time. This can be partially explained by elevated blood flow during the first imaging session in response to trauma from the surgery, which was also detected by OCT flow imaging. These results are promising for long-term optical access through the WttB implant, making feasible chronic *in vivo* studies in multiple neurological models of brain disease.

## Introduction

Repeated non-diffuse optical imaging of the brain is difficult. This is due to the fact that the cranial bone is highly scattering and thus a strong optical barrier. Repeated craniotomies increase the risk of complications and may disrupt the biological systems being imaged, motivating the development of several approaches to image the brain without repeatedly opening the skull. Modalities such as Magnetic Resonance Imaging (MRI), Computed Tomography (CT), Transcranial Doppler Ultrasonography, Positron Emission Tomography (PET), Single-photon Emission Computerized Tomography (SPECT), Near-Infrared Spectroscopy (NIRS), and Fluorescence Spectroscopy (with limited penetration depth) are capable of imaging through the intact skull (Amyot et al., 2015), but limited spatial resolution precludes more detailed studies of brain function and disease using these approaches. Other approaches have focused on mitigating scattering by the skull so that imaging strategies with higher spatial resolution can be applied to the brain. In animal models, these approaches have included thinning (Parthasarathy et al., 2010; Szu et al., 2012) or polishing (Shih et al., 2012) the skull, rendering the skull temporarily transparent using skull optical clearing agents (Wang et al., 2012a), or implanting temporary transparent windows made from glass or polymers (Roome and Kuhn, 2014; Zuluaga-Ramirez et al., 2015; Heo et al., 2016). These represent powerful scientific tools for studying the brain, but are not feasible for chronic imaging applications in human patients.

We previously introduced a potential solution (Castillo-Vega et al., 2012; Damestani et al., 2013, 2016a,b; Gutierrez et al., 2017; Davoodzadeh et al., 2018, 2019; Cano-Velázquez et al., 2019) in the form of a transparent ceramic cranial implant called the Window to the Brain (WttB) implant. This implant is made of nanocrystalline Yttria-Stabilized Zirconia (nc-YSZ), which possesses the requisite mechanical strength (Davoodzadeh et al., 2019) and biocompatibility (Damestani et al., 2016b) to serve as a permanent optical access window in human patients. In our previous work, we demonstrated the *in vivo* use of this WttB implant for Optical Coherence Tomography (OCT) imaging of the brain in mice (Damestani et al., 2013) as well as for repeated Laser Speckle Imaging (LSI) of mouse cerebral blood flow (Davoodzadeh et al., 2018) over 4 weeks. Our previous OCT study showed an improvement in imaging depth and signal-to-noise ratio (SNR) compared to imaging through unaltered mouse skull; however, the study was acute in nature and did not assess whether imaging quality across the WttB implant is maintained over time. Our LSI study showed that contrast and sharpness of brain vessels improved compared to imaging through the native mouse skull, but could not address the question of whether optical access remains stable over 4 weeks of imaging, as the cerebral blood vessels appeared to have different blood velocities and volumes at the different time points assessed. It was not possible to determine whether this was due to changes in the optical access provided by the WttB implant, or physiologic changes in the mouse between the different time points.

In this present study, we demonstrate repeated brain imaging of *n* = 5 mice using both OCT and LSI across the WttB implant over 4 weeks. The main objectives are to determine if the WttB implant allows for chronic OCT imaging, and to shed further light on the question of whether optical access provided by the WttB implant remains stable over this duration in the body.

## Materials and Methods

### Implant Fabrication and Preparation

The transparent 8 mol% YO_1.5_ nc-YSZ WttB implants used in this study were fabricated from a precursor yttria-stabilized zirconia nanopowder (Tosoh USA, Inc., Grove City, OH, USA) densified into a bulk ceramic via Current-Activated Pressure-Assisted Densification (CAPAD) as described previously (Garay, 2010). The resulting ceramic discs were 19 mm in diameter and 1 mm thick. The thickness was reduced to ~300 μm by polishing with 30 micron diamond slurry on an automatic polisher (Pace Technologies, Tucson, Arizona USA). The two faces were then polished using progressively finer abrasives (from 30 μm diamond slurry down to 0.2 μm colloidal silica slurry) to reduce light scattering by the implant surfaces and thus increase transparency. Next, thinned and polished discs were cut into rectangles of ~2.1 × 2.2 mm using a diamond lapping saw (DTQ-5, WEIYI, Qingdao, China), and the cut implants were cleaned via sonication in acetone followed by thorough rinsing in water.

### Animals

*N* = 5 8–12 week old C57Bl/6 male mice were purchased from Jackson Laboratory and housed under a 12-h light and 12-h dark cycle with ad libitum access to food and water. All experiments were approved by the University of California, Riverside Institutional Animal Care and Use Committee, and were conducted in accordance with the National Institutes of Health and Institutional Animal Care and Use Committee guidelines.

### Surgical Procedures and Post-surgical Monitoring and Care

Craniectomy surgery was conducted as previously described (Davoodzadeh et al., 2018). Briefly, mice were anesthetized with isoflurane inhalation (0.2–0.5%) and ketamine/xylazine (K/X) (80/10 mg/kg, i.p.), with additional anesthetic administered as necessary. Hair was removed from the scalp using clippers and depilatory cream, and ophthalmic ointment was placed over the eyes. Mice were then secured into stereotaxic frames to immobilize the head for surgery. The surgical site was sterilized with alternating application of betadine and 70% EtOH (3 times). WttB implants were also sterilized in 70% EtOH.

**Figure 1:**
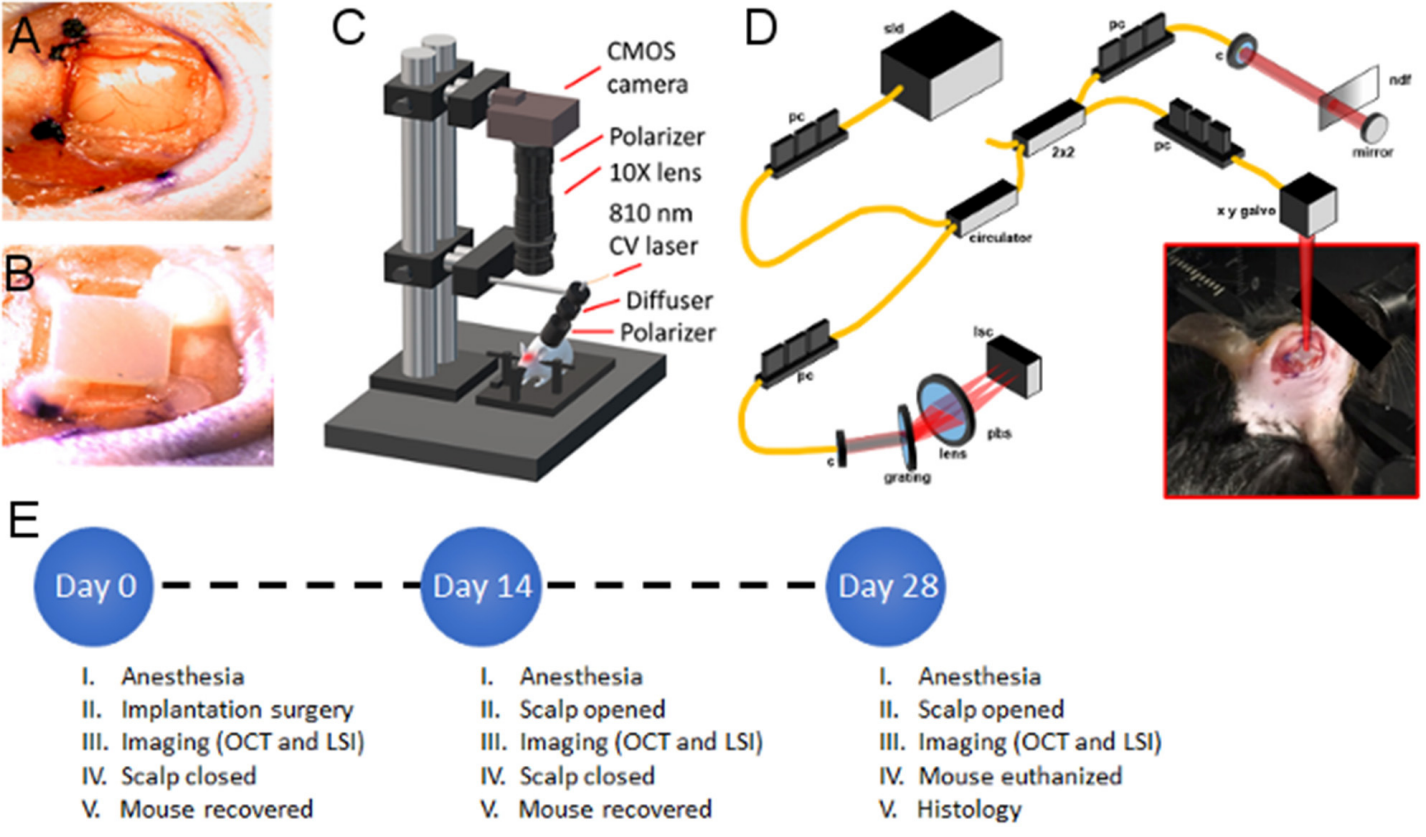
Experimental setup **(A)** craniectomy over right parietal lobe showing exposed Dura Mater, **(B)** WttB implant placement within craniectomy and fixation to skull with dental cement, **(C)** illustration of laser speckle imaging system, **(D)** illustration of optical coherence tomography imaging system, **(E)** timeline of surgical and imaging procedures for the *n* = 5 mice. sld, super-luminescent diodes; pc, polarization controller; c, collimator; pbs, polarization beam splitter; pm, polarization modulator; bs, beam splitter; ndf, neutral density filter; lsc, line scanning camera.

A sagittal incision was made to the left of the midline, and the scalp retracted to expose the skull. The periosteum was removed from the skull, and a craniectomy was performed with a surgical drill and carbide burr to remove a rectangular section of skull over the right parietal lobe, with dimensions slightly larger than the implant (Figure 1A). The YSZ implant was then placed within the craniectomy directly on the intact dura mater, and dental cement was applied to each of the four corners of the implant (to prevent displacement) and cured with blue light exposure for 20 s (Figure 1B).

The brain was imaged sequentially with LSI (Figure 1C) and OCT (Figure 1D) through the transparent WttB implant immediately after the cranioplasty, while the scalp was still open. Following the baseline imaging procedure, the scalp was closed with continuous suture. Triple antibiotic ointment was applied to the surgical site, and buprenorphine was administered (0.1 mg/kg, s.c.) for postoperative pain control. Mice were placed on a heating pad to aid recovery from anesthesia. A second buprenorphine injection was administered between 4 and 6 h after surgery.

For follow-up imaging, mice were anesthetized as described above, and the scalp was reopened to expose the implant. Periosteum overlying the implant was removed, and imaging was conducted in an identical manner to the baseline imaging. Following the final imaging procedure, mice were perfused, and brains were dissected and prepared for sectioning with a cryotome and histologic staining with Haemotoxylin and Eosin (H&E). A timeline of surgical procedures and imaging time points is provided in Figure 1E.

### Laser Speckle Imaging

The region of interest containing the WttB implant was illuminated by an 810 nm continuous wave laser (Vari-Lase REF946, Vascular Solutions, Morrisville, NC, USA) with incident power of 100 mW at a 45° incidence. The laser intensity was diffused and homogenized using an engineered diffuser (ED1-C20-MD; Thorlabs). A pair of negative-positive lenses (KPC043, −25 mm EFL and KPX094, 100 mm EFL; Newport, Irvine, CA) was used to expand the diffused laser light. The reflected light from the illuminated region was captured by a 12-bit complementary metal-oxide-semiconductor (CMOS) camera (DCC1545M; Thorlabs) equipped with a X10 zoom microscope (MLH-10X, 152.4 mm WD; Computar, Torrance, CA) focused at ~200 μm below the cortical surface. For each mouse over the time points, a sequence of 100 laser speckle images were captured at an exposure time of 6 ms [per our previous report on optimized LSI exposure time (Davoodzadeh et al., 2018)] at a speed of 14 frames per second. The aperture and magnification of the zoom microscope were carefully chosen to ensure that the speckle size at the image plane was approximate to the area of a single pixel in the CMOS chip (Cheng et al., 2003; Li et al., 2006, 2009). A schematic of the imaging system is shown in Figure 1C.

The laser speckle contrast images were calculated using temporal statistical analysis of laser speckle images. Experimental results have indicated that temporal speckle contrast analysis could expressively suppress the effect of the static laser speckle pattern formed by the stationary superficial reflection and scattering tissue on the visualization of blood flow (Cheng et al., 2003; Li et al., 2006, 2009; Zhu et al., 2010; Chen et al., 2016). Suppressing static laser speckle preserves spatial resolution and makes temporal contrast analysis an ideal method for imaging cerebral blood flow through the skull and implant The temporal contrast, *K*_*t*_, of each image pixel in the time sequence was calculated using Equation (1),

(1) $$\begin{array}{rcl} K_{t}\left(x,y\right) & = & \frac{\sigma_{\left(x,y\right)}}{\langle I_{\left(x,y\right)}\rangle }\\ = & \sqrt{\frac{1}{\left(N-1\right)}\left\{\overset{N}{\underset{n=1}{\sum }}{\left[I_{\left(x,y\right)}\left(n\right)-\langle I_{\left(x,y\right)}\rangle \right]}^{2}\right\}}\langle I_{\left(x,y\right)}\rangle \end{array}$$

where *I*_*x, y*_*(n)* is the CMOS counts at pixel (x,y) in the nth image, N is the number of images acquired, and < *I*_*x, y*_> is the mean value of CMOS counts at pixel (x,y) over the N images.

**Figure 4:**
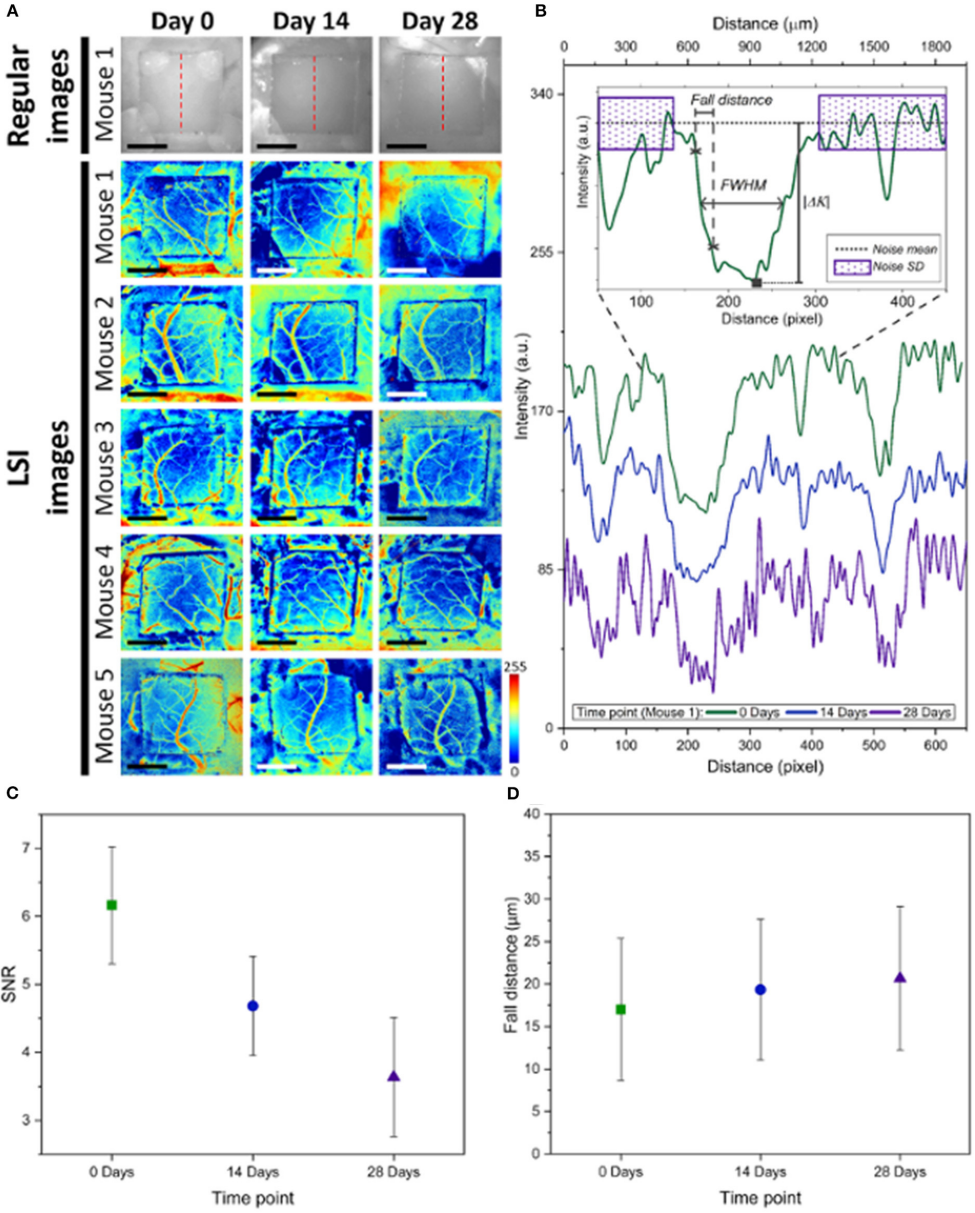
**(A)** Comparison of LSI images between Day 0, Day 14, and Day 28 for the *n* = 5 mice, as well as regular white light images of Mouse 1. The red dashed lines show where line profiles were taken at the midline of the WttB implant. **(B)** Analysis of line profiles from Mouse 1, showing decreasing intensity at days 14 and 28 compared t-o Day 0. The inset depicts how noise mean and standard deviation was measured, as well as fall distance and FWHM of vessels intersected by the line profiles. **(C)** Mean SNR for the 5 mice decreased at later time points compared to Day 0. **(D)** Fall distance of vessel edges increased at later time points compared to Day 0, indicating a reduction in the image sharpness at these later time points.

The quality of each speckle contrast image was assessed in terms of SNR and sharpness of the imaged vessels. To quantify signal to noise ratio for each imaging condition, the contrast intensity profile along a vertical line (across the blood vessels) was considered. To avoid selection bias, the location of these line profiles were chosen arbitrarily at the ROI mid-points (red dashed lines in Figure 4A). The midlines intersected 3 to 4 vessels, and remained the same between the time points. Figure 4B shows an example of the contrast intensity profile at day 0, 14 and 28 for Mouse 1. Equation (2) shows how SNR values were calculated for each time point using the contrast images,

(2) $$\begin{array}{r} SNR{=}^{\Delta K}{/}_{\sigma K_{n}} \end{array}$$

where Δ*K* is the depth of the vessel peak from the baseline (mean noise) and σ*K*_*n*_ is the standard deviation of the noise. For each time point, the SNR values were averaged over the mice and standard errors were calculated.

We compared the sharpness of the vessel edges in contrast images by calculating fall distance [the number of pixels multiplied by the pixel size (~3 μm)] of the edge of the vessel to go from 10 to 90% of Δ*K* value (Fauver et al., 2005). A shorter fall distance corresponds to greater sharpness. Fall distance was calculated for vessel edges intersected by the same line profiles used in the SNR calculation (red dashed lines in Figure 4A). Figure 4B shows an example trace and 10–90% fall distance measurement.

### Optical Coherence Tomography Imaging

Our initial investigation of the transparency of the nc-YSZ implant using OCT to image the brain of an acute murine model successfully demonstrated an increase in back reflected intensity and subsequent improvement of OCT image SNR (Damestani et al., 2013). The same laboratory-developed spectral-domain OCT (SD-OCT) system was used here to assess the chronic stability of image quality.

The core component of an OCT system is low coherence interferometry. The broadband laser light beam, typically in the near-infrared spectral range, is split into two optical paths, where there is a mirror at the end of one path and the sample at the end of the other. The interference pattern of the backscattered light from the two paths is measured, demodulated, and mathematically transformed into one line of depth-resolved image. By governing the position of the laser beam on sample, two-dimensional (2D) cross-sectional or three-dimensional (3D) volumetric images can be obtained. The spectral-domain OCT system used in this study utilizes a broadband laser light source with center wavelength of 1298 and 120 nm full-width at half maximum (FWHM). The total optical power from the source is 16 mW. The laser light is split between the reference arm and the sample arm at a beam splitter, and the returning light beams from each arm combine and interfere with each other resulting in coherent light. A diffraction grating at the detection portion of the system spreads the coherent light into spectral form and the spectrum is detected by a line scan camera (Sensors Unlimited, Princeton, NJ) with a line acquisition rate of 16.5 kHz. Each spectrum is converted to a spatial-domain depth-resolved A-line of image by applying inverse Fourier transform in post computational processing. Further details of the OCT system have been previously reported by Wang et al. (2012b).

**Figure 2:**
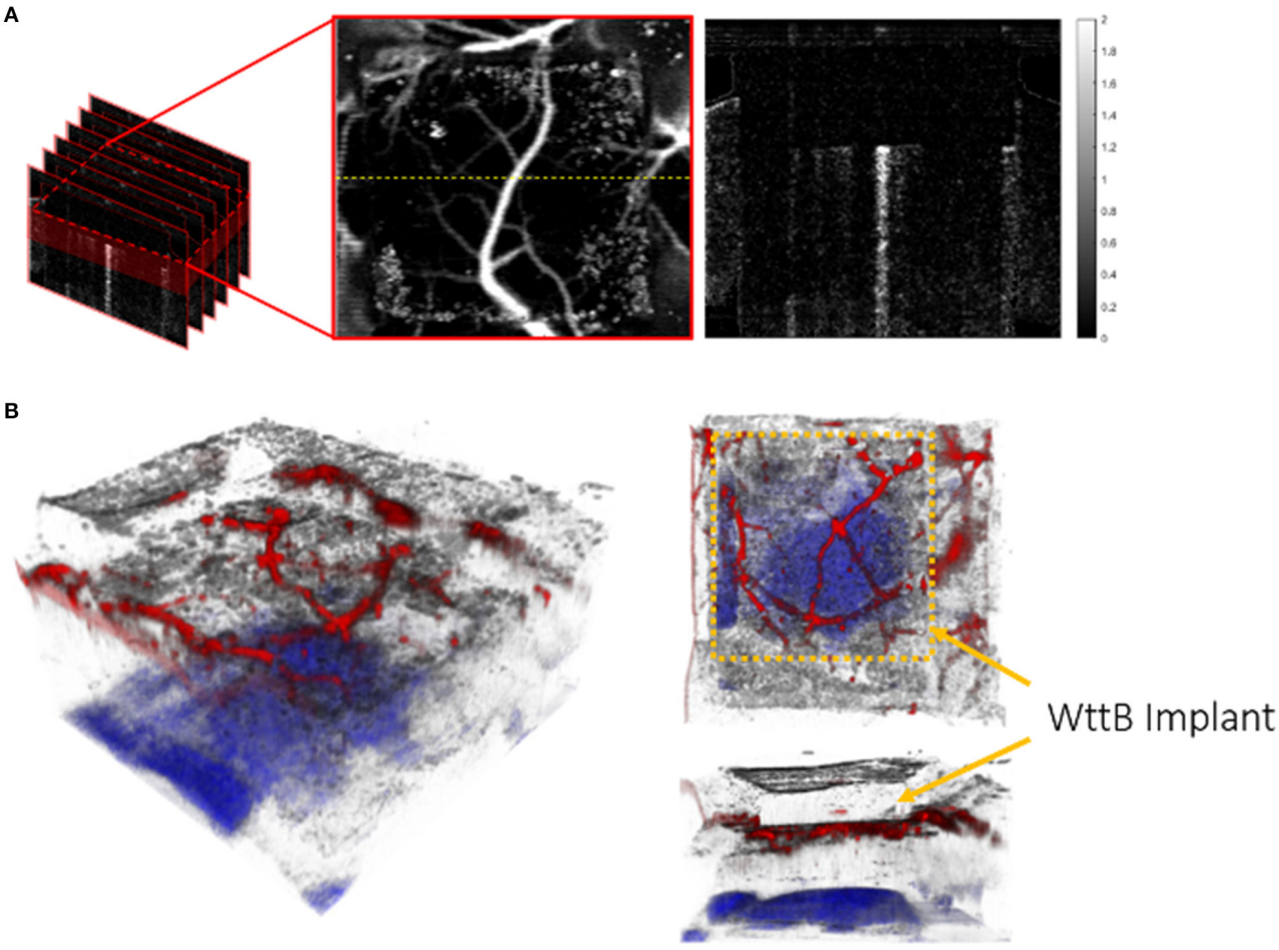
Schematic for volumetric and en face OCT visualization: **(A)** Volumetric flow information was constructed from 200 cross-sectional frames 2 mm in depth, covering a 3 mm by 3 mm square region laterally across the surface of the brain. As the majority of visible vessels occur within 0.4 mm beneath the lower surface of the WttB implant, only this depth range was used to create an en face view. The image on the right side is one example of cross-sectional flow image located at the yellow dashed line on the *en face* flow image (unit of the color bar: rad). **(B)** 3D visualization of OCT image of brain with WttB implant. Cerebral vasculature is colored in red and the corpus callosum, as identified through depth-resolved attenuation (Vermeer et al., 2013), is colored in blue. The upper and lower surfaces of the implant are visible, and can be contrasted with surrounding regions of native skull.

**Figure 3:**
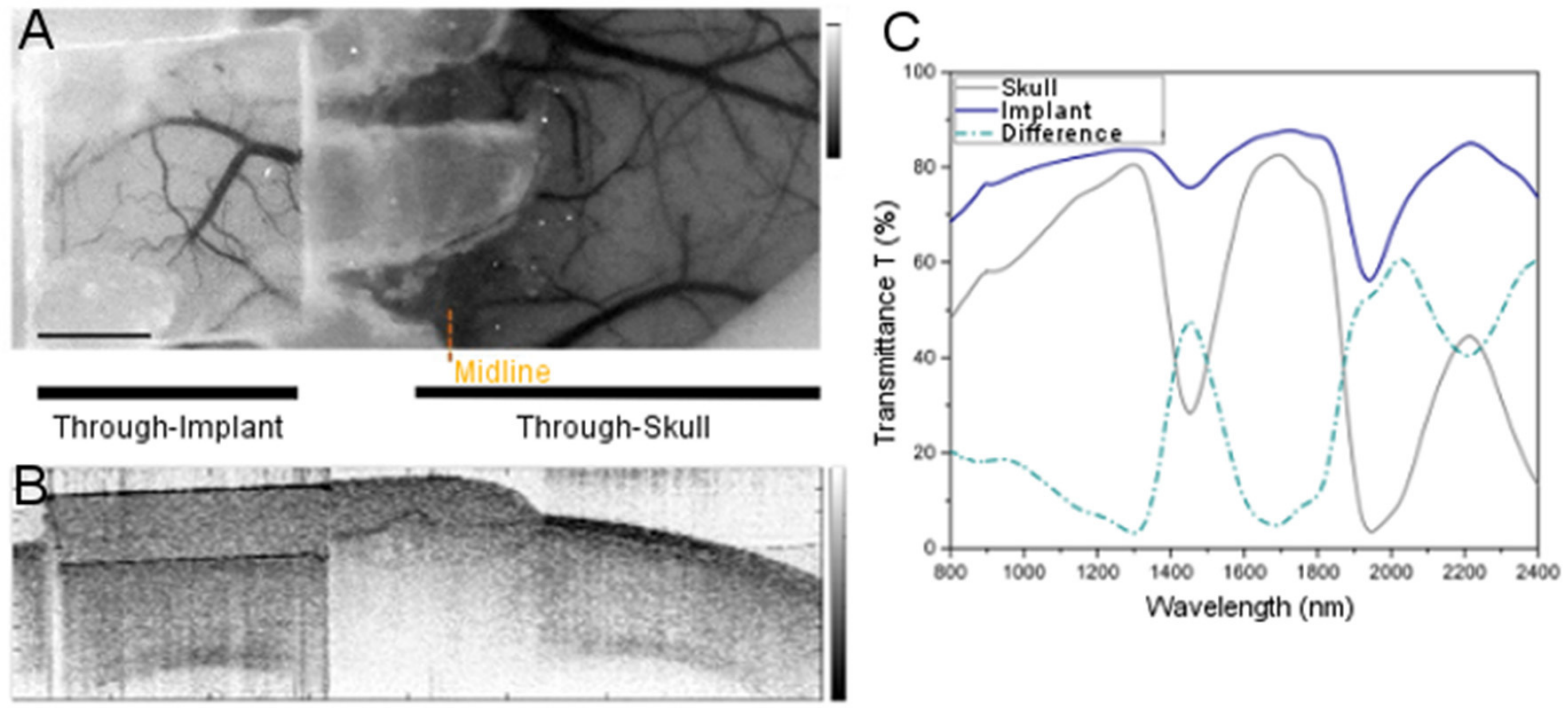
Comparison of imaging through WttB implant vs. native mouse skull. **(A)** LSI image of mouse cerebral vasculature with WttB implant, **(B)** OCT image of mouse cranium with WttB implant, **(C)** collimated transmittance spectra of the implant and mouse skull, showing the higher transparency of the implant compared to mouse skull.

**Figure 5:**
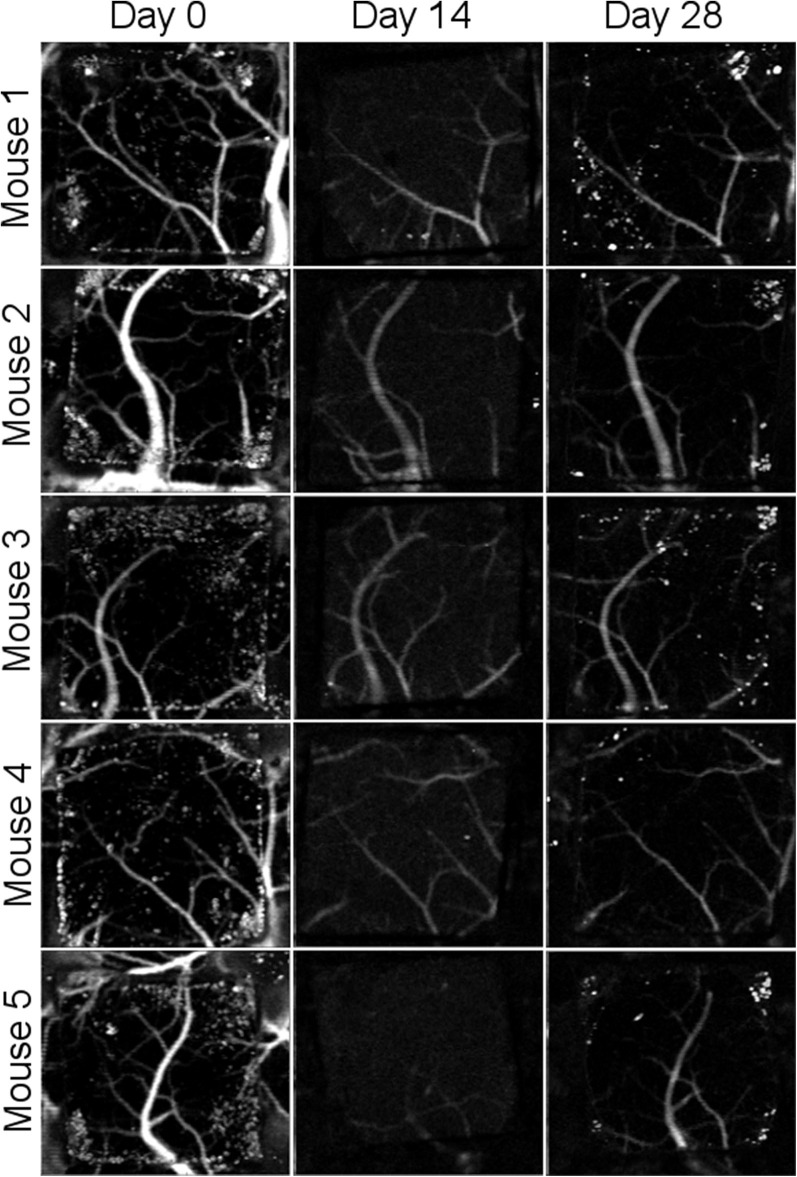
Comparison of *en face* OCT flow images between Day 0, Day 14, and Day 28 for the *n* = 5 mice. Flow appears higher at Day 0 than at later time points.

**Figure 6:**
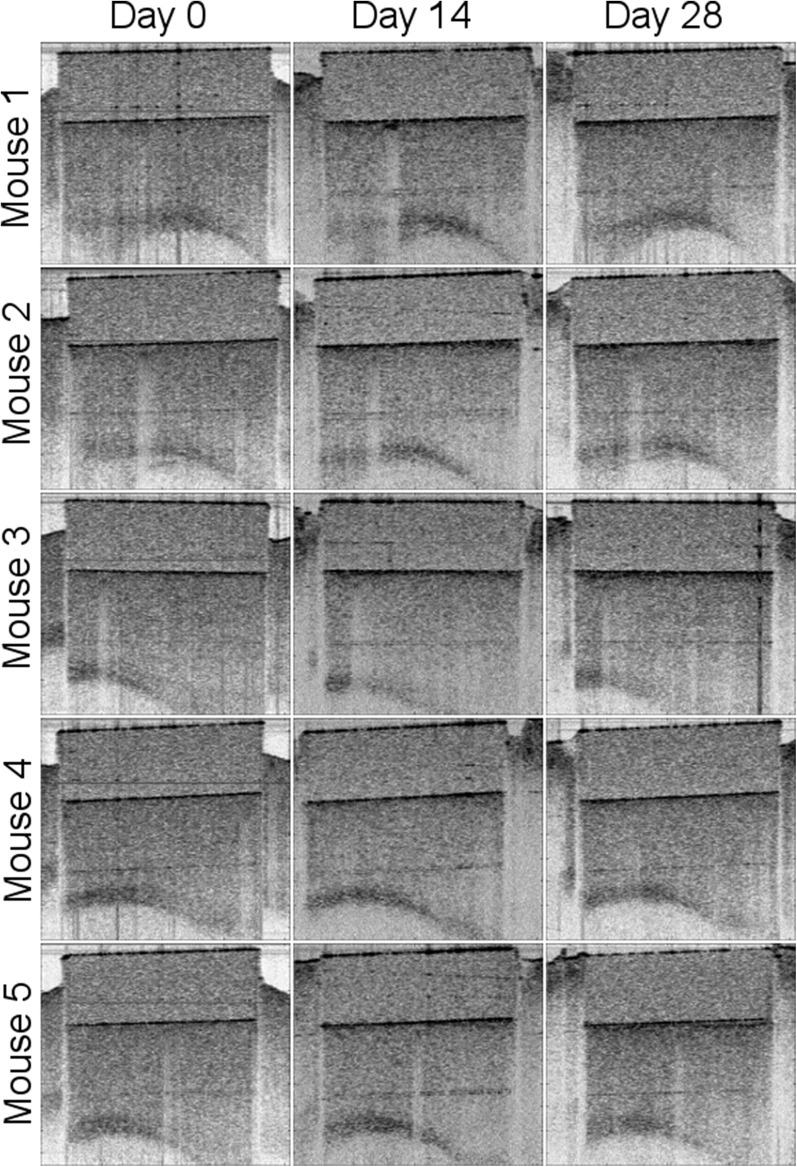
Comparison of cross-sectional OCT intensity images between Day 0, Day 14, and Day 28 for the *n* = 5 mice.

For all OCT imaging in this study, the OCT beam incident on the sample was focused at ~0.8 mm in depth along the incident axis (10 μm spot size) by a cemented achromatic doublet lens with 30 mm focal length (Thorlabs Inc., Newton, NJ). 2D cross-sectional images or 3D volumetric images were obtained by lateral scanning of the beam across the sample. Each of the cross-sectional OCT images presented in this study contain 2048 A-lines in a physical transverse range of 7 mm (Figure 3B) or 3 mm (Figure 6), whereas 3D volumes for *en face* vascular angiography analysis are composed of 200 cross-sectional frames, covering a 3 mm by 3 mm square region (Figures 2A, 5). In the previous and current study, the lateral resolution was ~ μm while the axial resolution was about 8 μm.

Real-time sample visualization and data acquisition was implemented by lab developed software with the aid of a graphics processing unit (GPU). Structural intensity images were processed and displayed in real time, allowing for rapid identification of the implant and location of the same region of the brain for all imaging sessions. The angle of incidence of the optical beam with respect to the WttB surface was fixed to ensure the consistency of results.

#### Cross-Sectional OCT Intensity Images

**Figure 7:**
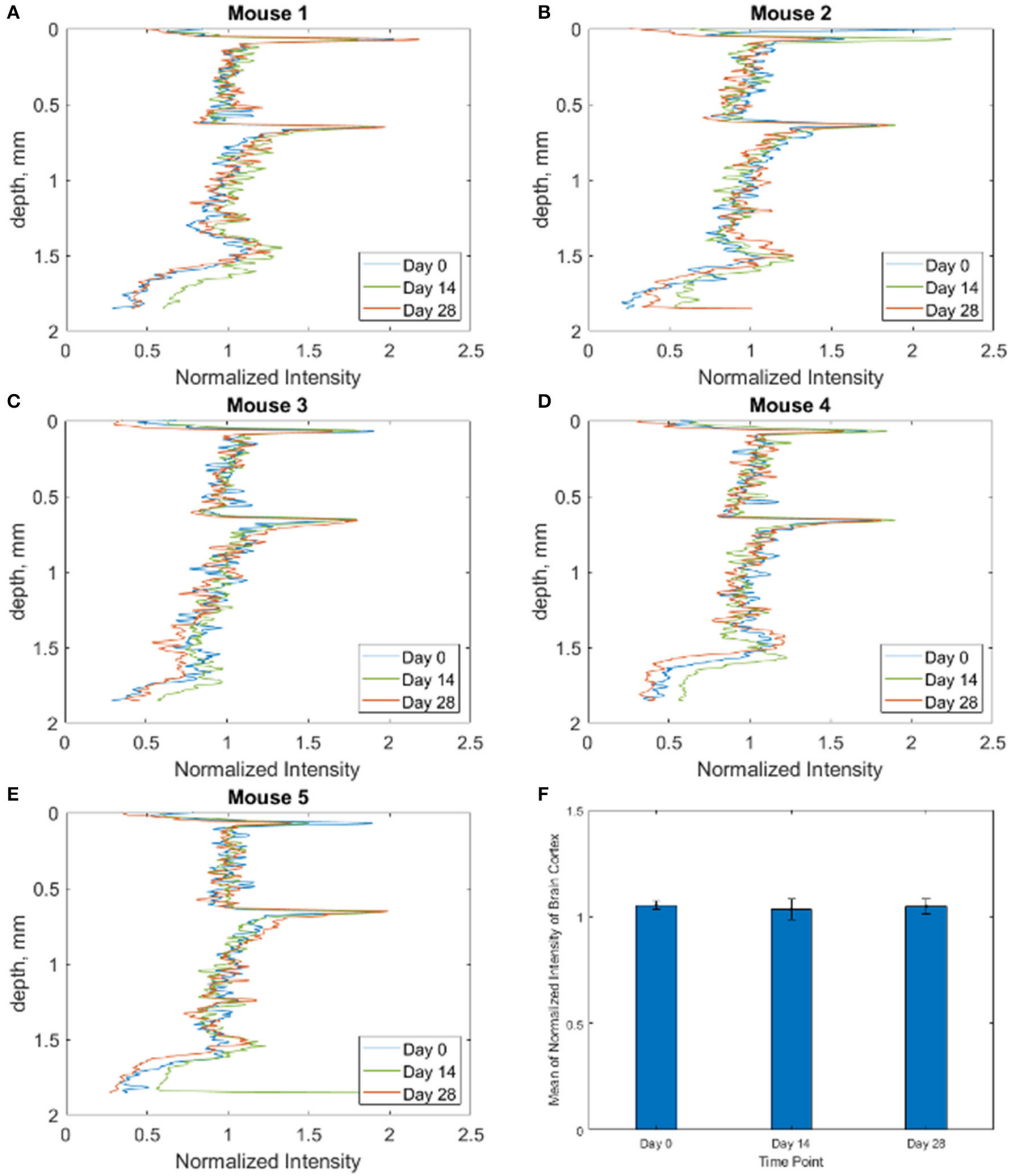
**(A–E)** Intensity depth profiles for each of the *n* = 5 mice at each time point, showing that attenuation is not increasing over the 28 days. **(F)** Bar graph showing mean of normalized intensity of brain cortex for all 5 mice at each time point.

A cross-sectional OCT intensity image (Figures 3B, 6) was formed by consecutive A-lines and displayed on a decibel scale. The blackness and whiteness represent the regions of high and low backscattered light intensity, respectively. Quantitative analysis of the light signal intensity (decibel scale) over image depth is presented in Figure 7. Averaged depth profiles were calculated by horizontally averaging 200 A-lines in the middle of the image that contains the information of WttB implant, cortex, and sub-cortical white matter (corpus callosum). The amount of signal level is a critical factor in the determination of image quality across the WttB implant. In our previous study, we concluded that the WttB implant enhanced imaging performance because the signal strength was significantly higher when imaging through the implant (Damestani et al., 2013). Similarly, in the assessment of imaging performance through the implant over time, we compare the image signal intensity of the same murine brain model taken at different time points over 4 weeks.

#### OCT Angiography Images

The motion of dynamic particles, such as red blood cells in vessels, can be determined by extracting temporal and spatial statistical information from OCT data. The detected OCT signals contain the superposition of back reflected components from static and dynamic scatterers, and OCT angiography techniques aim to screening out the dynamic scattering from static scattering for good visualization of vasculature and measurement of blood flow velocity (Ren et al., 2006; Tao et al., 2008; Srinivasan et al., 2012). Current OCT-based angiography techniques can be categorized into complex signal-based methods, phase-based methods and intensity-based methods (Baran and Wang, 2016; Zhu et al., 2017). Most of these techniques rely on the calculation of variance, decorrelation or a combination of both. The scanning protocol of OCT angiography typically requires high line acquisition rate and repeated scanning on the same location over a certain amount of time to obtain angiographic images with high sensitivity, however, due to the limitation of scanning speed of our system and limited time allowed for imaging because of animal anesthesia status, we generated the angiography images by computing the phase variance of two adjacent complex depth profiles.

As presented in our previous research (Hyle Park et al., 2005; Wang et al., 2012b; Baran and Wang, 2016; Zhu et al., 2017), phase variance is determined by the phase difference Δ(ϕ_*n*_) resulted from the comparison of the phases in one depth profile to those in the previous depth profile at corresponding depth (*z*_*m*_), yielding

(3) $$\begin{array}{r} {\Delta \phi }_{n}\left(z_{m}\right)=\phi_{n}\left(z_{m}\right)-\phi_{n-1}\left(z_{m}\right) \end{array}$$

where *n* is the depth profile index while *m* is the index of pixel in the depth profile. The minimal detectable phase difference (σ_Δϕmin_) is rudimentarily limited by the SNR of a measurement, which can be expressed as

(4) $$\begin{array}{r} \sigma_{\Delta \phi \min}=\frac{1}{\sqrt{SNR}} \end{array}$$

where *SNR* is the harmonic mean of the signal-to-noise ratio of the depth profiles involved in that measurement. Consistent sampling of data at the same location is fundamental to ensure that the measured phase difference is purely caused by the motion of flow. A correction algorithm to reduce the phase instability due to the movement of scanning mirror is included in the flow image processing (Pierce et al., 2002). Therefore, with the correction of overall phase shift between depth profiles, the phase variance can be calculated as

(5) $$\begin{array}{r} {\sigma_{\Delta \phi }}^{2}\left(z_{m}\right)={\left({\Delta \phi }_{n}\left(z_{m}\right)-\frac{\underset{z_{m}}{\sum }{\Delta \phi }_{n}\left(z_{m}\right)\cdot SNR{\Delta \phi }_{n}\left(z_{m}\right)}{\underset{z_{m}}{\sum }SNR{\Delta \phi }_{n}\left(z_{m}\right)}\right)}^{2} \end{array}$$

Spatial two-dimensional mean filter was applied to cross-sectional phase variance images to reduce the phase variance from arbitrary speckle noise. *En face* vasculature angiography was generated based on a depth range of 200 pixels (~ 1 mm) of subsurface cortex region below the window implant.

The 3D visualization of a wide view of the brain with WttB implant (Figure 2B) was constructed from a volumetric OCT imaging dataset with 800 frames and 4096 A-lines per frame. The same filtering strategy was applied to each frame of phase variance image and the depth of each blood vessel was determined by the measurement of phase variance along corresponding A-lines. For better visualization of the high-scattering brain structure, such as corpus callosum, we utilized the calculation of depth-resolved attenuation coefficient as described in Vermeer et al. (2013). The processed sets of phase variance images and attenuation coefficient images were imported to Amira (Thermo Fisher Scientific and Zuse Institute Berlin) for 3D rendering.

## Results

Figures 3A,B provide LSI and OCT images with a wide field of view covering both sides of the head, to allow for visual comparison of brain vasculature and structural imaging, respectively, through the WttB implant vs. the skull. Figure 3C shows the near-infrared collimated transmittance spectra of the implant vs. the mouse skull, including the wavelengths used for LSI (810 nm) and OCT (1238–1358 nm) imaging.

Figure 4A shows the LSI images of cerebral blood flow for each of the 5 mice at the 3 time points of this study. The images are displayed as color maps, where higher relative velocity within the image (i.e., regions with blood flow) appear red and lower relative velocity within the images (i.e., static regions of tissue) appear blue. Figure 4A also includes regular white light images of Mouse 1. For all LSI images, line intensity profiles were taken at the midpoint of the implant (illustrated as red dashed lines in the regular white light images) for quantitative analysis. Figure 4B shows the line intensity profiles for Mouse 1 at the 3 time points, which allowed for identification of vessels intersected by the line profile which appear as valleys in the intensity profile. The line profiles further allowed for determination of SNR, and fall distance of the vessel edges (see inset). Mean SNR and fall distance for the 5 mice are provided for each time point in Figures 4C,D, respectively (error bars represent standard error).

LSI alone was not sufficient to answer the question of whether optical access provided by the WttB implant remains stable, as blood flow does not remain constant over time and differences found between the imaging time points could be due to physiologic changes such as hyperemia, changes in the optical access, or a combination of both. To answer this question, we used OCT imaging, and processed the data to render *en face* flow images (which provide similar information to LSI), and cross-sectional intensity images (which provide structural information on tissue and should remain stable over time). Figure 5 presents the OCT *en face* flow images for the 5 mice at the 3 imaging time points, and is in general agreement with the LSI images, showing higher flow at Day 0 compared to the later time points.

Cross-sectional OCT intensity images are shown in Figure 6 for the five mice across the three time points. All OCT images were acquired to position in the WttB implant within the same depth range to minimize the effect of depth-dependent OCT sensitivity between imaging sessions. The images were further normalized by the average intensity from within the interior of the Wttb implant to further reduce the effects of slight variations in experimental acquisition between imaging sessions, such as the angle of incidence with respect to the implant surface. Normalized intensity depth profiles for each mouse at each time point are provided in Figure 7. The degree of overlap between these depth profiles provides quantitative verification of the longitudinal stability of OCT imaging through the WttB implant over the 28 day period.

**Figure 8:**
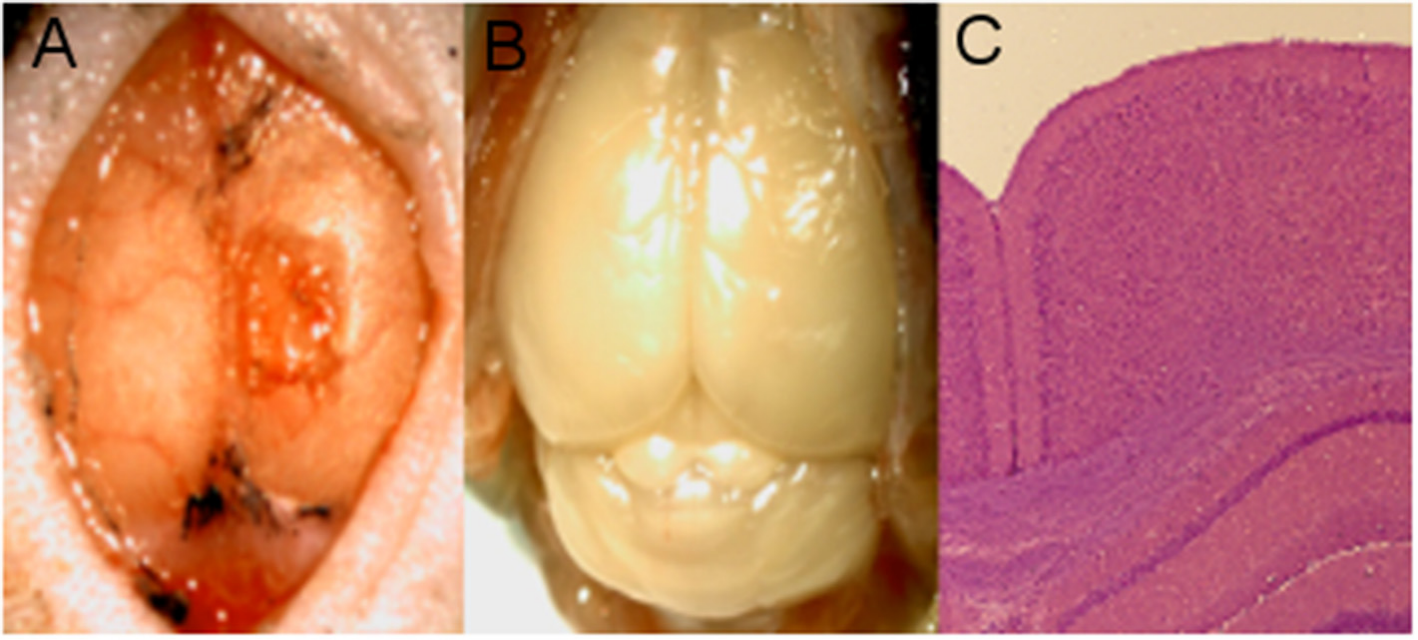
Mouse 1 histology. **(A)** Craniectomy with implant removed following euthanasia. **(B)** Brain with skull removed. **(C)** H&E stain of brain section from beneath the implant, showing no signs of inflammation.

Following the final imaging time point on Day 28, mice were sacrificed and histology was performed on the brain via H&E staining. Cross-sectional sections of the brain beneath the craniectomy showed no signs of inflammation or histological damage. An example from Mouse 1 is provided in Figure 8.

## Discussion

The Window to the Brain implant seeks to provide ongoing optical access to the brain, on demand, without the need to repeated craniectomies to open the skull. In this study, the scalp was opened for each imaging session to allow for direct comparison of optical access across the implant at each time point, without contribution from the scalp. In the envisioned clinical implementation, the scalp would not be reopened for imaging sessions. Instead, scalp overlying the implant would be rendered temporarily transparent through topical application of optical clearing agents (OCAs). We have demonstrated this strategy with the WttB implant previously, using *ex vivo* skin samples (Cano-Velázquez et al., 2019). While the implant could be attached directly to the skull, an alternative possibility would be to integrate this window into existing cranial implants (e.g., PEEK, PEKK, or Ti plates) as a viewing port. This would allow for existing surgical implantation protocols to be used, and would ease the clinical translation of these windows. As with all cranial implants, a major concern is infection of the implant during and after implantation. In the case of existing opaque implants, removal and replacement of the fouled implant is the current standard of care when infection occurs. The transparency of the WttB implant provides the potential to perform laser antifouling of both upper and lower implant surfaces *in situ*, which could prevent the need for these additional open-skull procedures. We have demonstrated this concept previously *in vitro* in cultures of E. coli (one of the primary causes of conventional implant failure) (Damestani et al., 2016a).

Consistent with our previous studies (Damestani et al., 2013; Davoodzadeh et al., 2018), imaging through the WttB implant with LSI and OCT resulted in higher contrast than imaging through the mouse skull (Figures 3A,B, respectively). Collimated transmittance spectra for the skull and WttB implant are provided in Figure 3C, and show that transmittance is higher through WttB implant than skull for all wavelengths measured, including 810 nm (LSI wavelength) and 1298 nm (OCT central wavelength). It should be noted that although the difference in transparency between the implant and mouse skull is modest for some wavelengths, the mouse skull is inherently transparent (Li et al., 2006), which is not the case for human skull where the difference between implant and skull transmittance will be larger.

In our previous study (Davoodzadeh et al., 2018), LSI imaging of cerebral blood flow across the WttB implant did not remain constant over the 4 weeks. SNR and sharpness of the imaged vessels were found to be decreased at Day 14 and Day 28 compared Day 0. Visually, the vessels appeared to have smaller diameters and lower relative blood flow velocity at these later time points. The data presented in this prior study came from 3 of the 5 mice presented in this present work, from data acquired at these same imaging sessions. Figure 4 includes data from these mice along with 2 additional mice, which show the same trend of decreased SNR and sharpness of imaged vessels at later time points compared to Day 0 imaging. In the previous study, we were unable to answer whether the optical access provided by the WttB implant was degrading over time, or whether the blood flow was different at the different imaging time points. We speculated that the change we observed was primarily due to changing blood flow and not to a change in the optical access, and that blood flow was elevated above normal levels on Day 0 due to the craniectomy surgery. This phenomenon of elevated blood flow in response to tissue trauma is known as reactive hyperemia (Gourley and Heistad, 1984). In this present study, the inclusion of OCT imaging provides us with additional information to answer this question. First, we sought to assess blood flow between the time points using OCT to see if it was in agreement with the LSI. As shown in Figures 4, 5, the OCT is in general agreement with the LSI, showing the vessels to be of larger diameter at Day 0, and with higher flow velocity, indicating that blood flow is indeed higher at Day 0 compared to Days 14 and 28.

To answer the question of whether the optical access to the brain provided by the WttB implant remains stable over time, we compared OCT intensity images between the 3 time points for each mouse. As shown in Figure 6, the images appear very similar over the 4 weeks of implantation. As shown in Figure 7, the intensity profiles of brain tissue imaged through the implant overlap and do not show any consistent trend toward faster intensity drop-off with depth between the time points, indicating that the attenuation of light collected through the implant is not increasing over the 4 weeks of this study.

Together, these data suggest that the WttB implant provides stable optical access to the underlying tissue, at least for the wavelengths assessed by OCT (1238–1358 nm). The changes we found in this study and our previous LSI study regarding vessel diameter and relative flow velocity are likely actual changes in the blood flow and not imaging artifacts, as supported by OCT when processed for *en face* flow information. The blood flow appears to be elevated at Day 0 (following the craniectomy), and this change is no longer present by Day 14, where the flow is much closer to Day 28 blood flow. The histology of brain slices taken beneath the implant (Figure 8) further support this interpretation of the data, showing no signs of inflammation or structural damage to the surface of the cortex.

There are several important limitations to the current study. While OCT angiography showed differential blood flow at the various time points in agreement with LSI, direct assessment of the blood flow (e.g., via injection of a contrast agent) was not performed and could have provided more direct validation of this interpretation of the imaging data. Similarly, histology of the cerebral cortex showed a lack of inflammation in response to the implant, but more detailed histology of the cerebral vasculature was not performed. Finally, our interpretation of the OCT intensity data is that attenuation of light through the implant is not increasing, and therefore the implant is not deteriorating over these 4 weeks in the body. This claim would have been strengthened by comparison of the optical and mechanical properties of the implant before implantation and after 4 weeks in the body. However, these optical and mechanical comparisons were made in a prior study (Davoodzadeh et al., 2019) where we simulated the aging that the implants would undergo over many decades in the body using ISO standard methods for the aging stability of zirconia implants (i.e., autoclave processing at 134°C at a water partial pressure of 2–3 bar; ISO 13356:2008) (Deville et al., 2006; Chevalier et al., 2007). This study showed that the WttB implant has excellent stability against low temperature degradation and did not exhibit any crystallite phase change, nor change in optical transmittance or Vickers hardness due to these tests, making such changes very unlikely over the 4 weeks of this current study.

## Conclusion

The Window to the Brain implant allowed for stable repeated imaging of the mouse brain with Optical Coherence Tomography over 28 days, without loss of signal intensity. Repeated Laser Speckle Imaging was also possible over this timeframe, but signal to noise ratio and the sharpness of vessels in the images decreased with time. This can be partially explained by elevated blood flow during the first imaging session in response to trauma from the surgery, which was also detected by OCT flow imaging. These results are promising for long-term optical access through the WttB implant, making feasible chronic *in vivo* studies in multiple neurological models of brain disease.

## Data Availability Statement

The datasets generated for this study are available on request to the corresponding author.

## Ethics Statement

The animal study was reviewed and approved by University of California Riverside Institutional Animal Care and Use Committee.

## Author Contributions

Implant preparation and surgical procedures were performed by DH, CJ, and PE. Optical coherence tomography imaging was performed by JL. Laser speckle imaging was performed by ND. Collimated transmittance measurements of implant and skull was performed by MC-V. Guidance on study design and data interpretation was provided by HP, DB, and GA. All authors contributed to manuscript revision, read and approved the submitted version.

## Conflict of Interest

The authors declare that the research was conducted in the absence of any commercial or financial relationships that could be construed as a potential conflict of interest.
